# Influence of time elapsed from end of emergency surgery until admission to intensive care unit, on Acute Physiology and Chronic Health Evaluation II (APACHE II) prediction and patient mortality rate

**DOI:** 10.1590/S1516-31802005000400003

**Published:** 2005-07-07

**Authors:** Paulo Antonio Chiavone, Samir Rasslan

**Keywords:** APACHE, Intensive care units, Prognosis, Time factors, Hospital mortality, APACHE, Unidades de terapia intensiva, Prognóstico, Fatores de tempo, Mortalidade hospitalar

## Abstract

**CONTEXT AND OBJECTIVE:**

Patients are often admitted to intensive care units with delay in relation to when this service was indicated. The objective was to verify whether this delay influences hospital mortality, length of stay in the unit and hospital, and APACHE II prediction.

**DESIGN AND SETTING:**

Prospective and accuracy study, in intensive care unit of Santa Casa de São Paulo, a tertiary university hospital.

**METHODS:**

We evaluated all 94 patients admitted following emergency surgery, from August 2002 to July 2003. The variables studied were APACHE II, death risk, length of stay in the unit and hospital, and hospital mortality rate. The patients were divided into two groups according to the time elapsed between end of surgery and admission to the unit: up to 12 hours and over 12 hours.

**RESULTS:**

The groups were similar regarding gender, age, diagnosis, APACHE II score and hospital stay. The death risk factors were age, APACHE II and elapsed time (p < 0.02). The mortality rate for the over 12-hour group was higher (54% versus 26.1%; p = 0.018). For the over 12-hour group, observed mortality was higher than expected mortality (p = 0.015). For the up to 12-hour group, observed and expected mortality were similar (p = 0.288).

**CONCLUSION:**

APACHE II foresaw the mortality rate among patients that arrived faster to the intensive care unit, while the mortality rate was higher among those patients whose admission to the intensive care unit took longer.

## RESUMO

**CONTEXTO E OBJETIVO:**

Com freqüência os doentes são admitidos nas unidades de terapia intensiva com atraso em relação ao momento da indicação deste atendimento. Objetivamos verificar, nos doentes operados de urgência, se esse atraso interfere na letalidade hospitalar, na permanência na unidade e no hospital, e na predição do escore APACHE II.

**TIPO DE ESTUDO E LOCAL:**

De acurácia, prospectivo, na unidade de terapia intensiva da Santa Casa de Misericórdia de São Paulo, hospital terciário e universitário.

**MÉTODOS:**

Foram avaliados todos os 94 doentes admitidos de agosto/2002 a julho/2003, após operações de urgência. As variáveis estudadas foram: APACHE II, risco de óbito, permanência na unidade e no hospital e letalidade hospitalar. Foram comparados dois grupos de doentes divididos conforme o intervalo de tempo entre o fim da operação e a admissão na unidade: até 12 h e após 12 h.

**RESULTADOS:**

Os grupos eram semelhantes quanto ao sexo, idade, diagnóstico, APACHE II, tempo de internação, bem como de hospitalização. Os fatores de risco de óbito foram idade, APACHE II e tempo de retardo na internação (p < 0,02). A letalidade do grupo após 12 h foi maior (54.9% × 26.1%; p = 0,018). A letalidade observada foi maior que a esperada no grupo após 12 h (p = 0,015), enquanto a letalidade observada no grupo até 12 h foi semelhante à esperada (p = 0,288).

**CONCLUSÕES:**

O APACHE II previu melhor a letalidade do grupo de doentes que entraram mais rapidamente na unidade de cuidados intensivos, enquanto a letalidade hospitalar foi maior nos doentes que demoraram mais para a admissão na unidade de terapia intensiva.

## INTRODUCTION

The intensive care unit is the part of the hospital that is dedicated to providing a system of continuous surveillance for seriously ill patients who are potentially recoverable or at risk.^1^ The high-complexity features of these services and their high cost make it difficult to offer enough beds in intensive care units to cope with the growing demand. This demand is a reflection of increased life expectancy, longer survival of patients with diseases that used to be lethal, progress in diagnosing and treating various diseases, introduction of new therapeutic procedures and better approaches, and pre-hospital treatment. All these factors bring into hospitals larger number of traumatized patients who would previously have died at the scene of the accident or on the way to hospital.

The need to adapt the number of intensive care unit beds for this growing demand has made evaluation of the prognosis an important aspect of clinical investigation. In Brazil, the prognostic index most used is APACHE II (Acute Physiology And Chronic Health Evaluation II),^2^ which has been evaluated and validated for use in several countries.^3-12^ Many Brazilian studies have evaluated its application to intensive care units, and APACHE II has been approved for use in quantifying the severity of patients' conditions. On the other hand, its calibration for predicting hospital mortality has not been good, and it is recommended that safeguards regarding differences between studied populations, correction factors and standard mortality rate should also be taken into consideration.^13-20^

Because APACHE II is the most used index, the need to evaluate the possible influences on its forecasting capacity continues to have significant importance. One of the factors that certainly interferes in the evaluation of Brazilian intensive care units is the great difference between the demand for high-complexity services and the limited capacity to absorb such patients.^20-22^ The lack of vacant beds, in conjunction with real need, especially in acute emergency cases, creates a situation in which, after completion of the surgical intervention, patients stay in the surgical unit awaiting transfer to the intensive care unit. This may have implications for the quality of treatment and, consequently, on such patients' evolution and prognosis.

## OBJECTIVE

To verify whether the elapsed time before transfer from the operating theater to the intensive care unit interferes in the predictive accuracy of the APACHE II index, length of stay in the intensive care unit and hospital, and hospital mortality, among patients who have undergone emergency surgery.

## METHODS

All the patients admitted to the intensive care service of Hospital Santa Casa de Misericórdia de São Paulo between August 2002 and July 2003 were evaluated. This is a tertiary teaching hospital with approximately 820 active beds, of which 600 are for adults. At the time when this study was conducted, the intensive care unit had 15 beds for clinical or surgical adult patients. The study protocol had previously been submitted for consideration by the institution's research ethics committee and had been approved.

We selected 104 patients admitted to the intensive care unit directly from the surgical unit, following emergency surgery. Of these, five patients were excluded because they had not come from the hospital's emergency service, i.e. they had already been hospitalized in other units of the hospital. Four patients were excluded because they had already been in the intensive care unit during the same hospitalization. One other patient was also excluded because some of the information needed for the study was missing. The evaluation started after admission to the intensive care unit, and thus there was no interference from the hospitalization criteria. The patients were prospectively followed up until death or hospital discharge. Retrospective evaluation was undertaken to obtain data regarding the period prior to admission to the intensive care unit. The patients were classified in one of two groups, according to the elapsed time between the end of the operation and the admission to the intensive care unit. The "up-to-12 h" group waited for up to 12 hours before admission, and the "over-12 h" group waited for more than 12 hours.

The 94 patients finally included in the study were evaluated with regard to the following information: gender, age, diagnosis, APACHE II, risk of hospital death, length of stay in the intensive care unit, length of hospital stay, and evolution until hospital discharge or death. The APACHE II index was calculated within the first 24 hours following admission to the intensive care unit. The expected mortality was calculated by the APACHE II system and was compared with the observed mortality. The standardized mortality rate was thus obtained by means of dividing the observed by the expected mortality. The death risks were compared with the observed mortality. The sensitivity, specificity and percentage of correct classification were calculated for all of the risk levels. A single observer did the data checking and processing.

### Statistical analysis

Regression analysis was performed to correlate between mortality and the time elapsed between the end of the operation and admission to the intensive care unit. Student's t test was used for comparing the averages of continuous measurements. The chi-squared test, with the Yates correction, was used for comparing the proportions of categorized measurements, except in subgroups with five patients or less, in which case Fisher's exact test was used. The chi-squared result was used as the trend. The regression analysis was performed to identify the risk factors for death. The predictive capability of the APACHE II index was assessed using the receiver operating characteristic curve, through a 2 x 2 decision matrix and linear regression analysis.

The significance level of p < 0.05 was considered statistically significant. The Statistical Package for the Social Sciences software, version 10.01, and Epi Info version 6.04 were used.

## RESULTS

Among the 94 patients included in the study, 23 (24.5%) were admitted to the intensive care unit within 12 hours after the end of the surgery, and these 23 patients formed the up-to-12 h group. The majority of the patients (71; 75.5%) were in the over-12 h group. There was male prevalence in both groups, but no significant difference in gender between the groups (p = 0.8539). With regard to age, in the up-to-12 h group it ranged from 16 to 76 years, with an average of 48 years (standard deviation, SD = 17), while in the over-12 h group it ranged from 18 to 97 years, with an average was 56 years (SD = 19), without significant difference between the groups (p = 0.0920).

**Figure 1:**
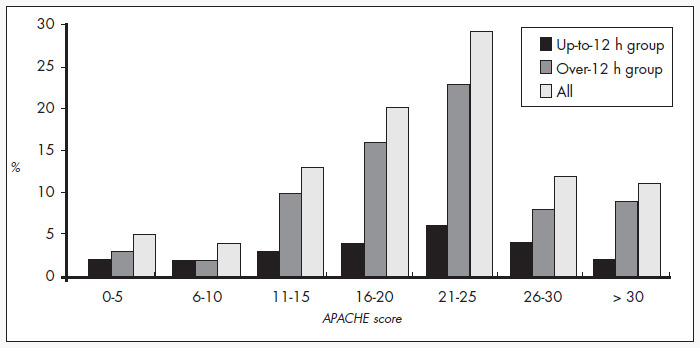
Concentration of patients according to their APACHE (Acute Physiology and Chronic Health Evaluation) score and to the time elapsed until admission to the intensive care unit.

The patient distribution in the diagnosis categories according to the APACHE II classification is shown in Table 1. There was no difference between the groups regarding the most frequent diagnoses, or in the numbers of patients in each category. The APACHE II score ranged from 2 to 41, with an average for the up-to-12 h group of 20.1 (SD = 9.6), while in the over-12 h group it was 21.1 (SD = 7.8), without significant difference between them (p = 0.6129). The greatest concentration of patients (52%) was in the APACHE II range of 16 to 25 (Figure 1).

**Table 1:** Distribution of 94 Brazilian intensive care unit patients at Hospital Santa Casa de Misericórdia de São Paulo, admitted from August 2002 to July 2003, according to diagnostic categories from Knaus et al.^2^ (1985)

Diagnostic Categories	Up-to-12 h group		Over-12 h group		Chi-squared	p
	n	%	n	%		
Multiple trauma	9	39.1	23	32.4	0.35	0.55
Sepsis and infection	5	21.7	15	21.1	0.05	0.82
Abdominal surgery	4	17.4	14	19.7	00.0	0.95
Cardiovascular surgery	1	4.3	10	14.1	0.79	0.37
Head trauma	1	4.3	5	7.0	0.00	0.95
Cerebrovascular insufficiency	1	4.3	4	5.6	0.09	078
Respiratory insufficiency following surgery	1	4.3	–	–	–	–
Medullar compression/ laminectomy	1	4.3	–	–	–	–
**Total**	**23**	**100**	**71**	**100**		

**Figure 2:**
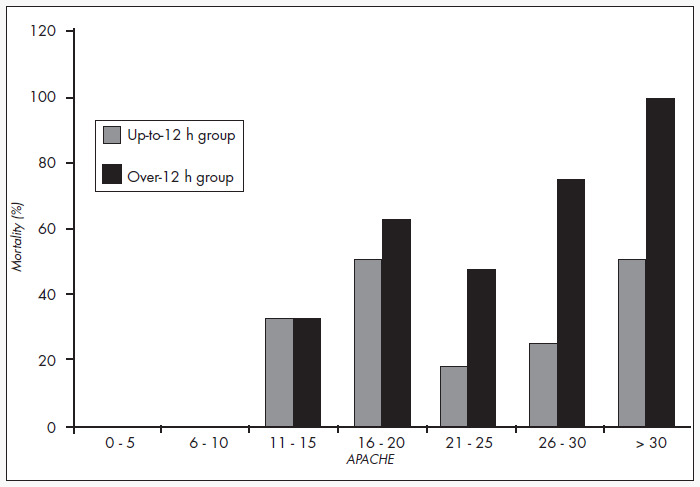
Mortality distribution of operated patients according to their APACHE (Acute Physiology and Chronic Health Evaluation) score and to the time elapsed until admission to the intensive care unit.

The general mortality was 47.9%, and the mortality in the over-12 h group was significantly greater than for the up-to-12 h group (54.9% versus 26.1%; p = 0.018). The observed general mortality was similar to the expected mortality. For the patients in the up-to-12 h group, the observed mortality was similar to the predicted mortality, while for the over-12 h group, the observed mortality was greater than the expected mortality (54.9% versus 39.7%; p = 0.015). The overall standardized mortality rate was 1.21, while it was 0.69 for the up-to-12 h group and 1.36 for the over-12 h group, as shown in Table 2. Figure 2 displays the mortality distribution in relation to the APACHE II ranges for the two groups.

**Table 2:** Observed death rate, predicted death rate and standard mortality rate for the two elapsed time groups, among 94 intensive care patients at Hospital Santa Casa de Misericórdia de São Paulo, from August 2002 to July 2003

Groups	Observed death rate %	Predicted death rate %	p*	SMR
Up-to-12 h	26.1	37.9	0.288	0.69
Over-12 h	54.9	40.3	0.015	1.36
Total	47.9	39.7	0.11	1.21
p**	0.016			

*SMR = s tandardized mortality rate;*

* *Binomial comparison between observed and predicted death rate for each group;*

** *Chi- squared = 5.79; comparison between observed death rate in up-to-12 h versus over-12 h groups.*

The average length of stay in the intensive care unit was 14.7 days for the up-to-12 h group and 12.8 days for the over-12 h group, without statistical difference between them (p = 0.9077). The average length of hospital stay was 39.0 days for the up-to-12 h group and 28.1 days for the over-12 h group, without statistical difference between them (p = 0.4462).

**Figure 3:**
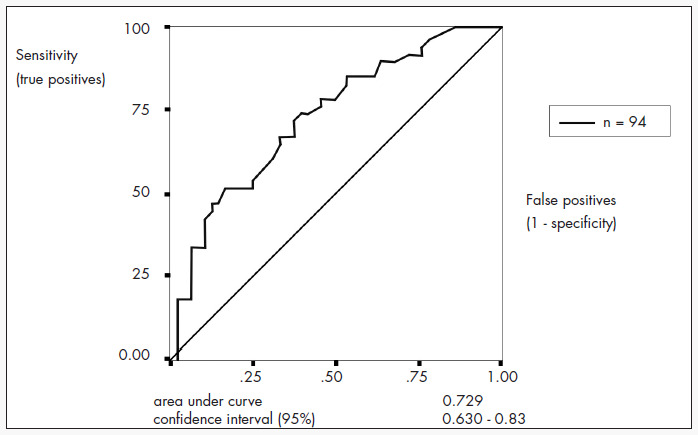
Receiver operating characteristic (ROC) curve for risk of death as predicted by Knaus equation among 94 patients in the intensive care unit of Santa Casa de Misericórdia Hospital in São Paulo, Brazil, between August 2002 and July 2003.

**Figure 4:**
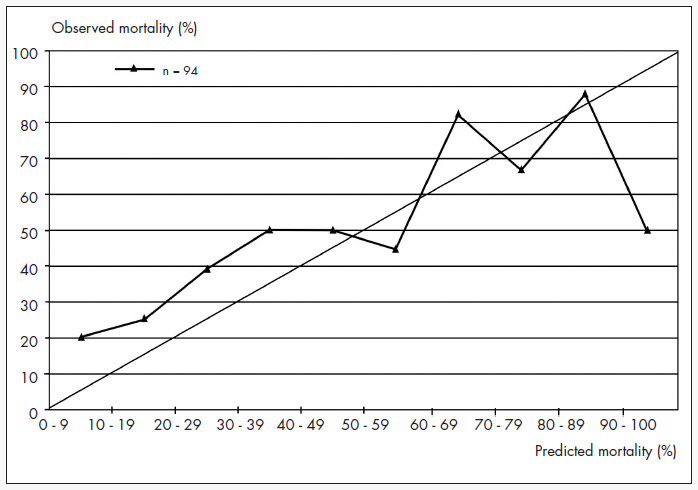
Calibration curve (observed mortality x predicted mortality) for 10% risk intervals among 94 patients in the intensive care unit of Santa Casa de Misericórdia Hospital in São Paulo, Brazil, between August 2000 and July 2003.

The death risk factors identified through the regression logistic analysis were: age, APACHE II, elapsed time before admission to the intensive care unit and length of hospital stay (p < 0.02). The receiver operating characteristic curve, built up from the sensitivity and the complement of the specificity of the death risk, shows an area under the curve of 0.729 (Figure 3). The best percentage of correct classification was obtained with a decision criterion of 0.6, and was 67%. The calibration curve, stratified in 10% risk bands, obtained an r2 value of 0.46 (Figure 4).

## DISCUSSION

Even in the more developed countries, the complexity and the high cost of intensive care services make it difficult for hospitals to have enough intensive care beds for the growing patient demand. In the United Kingdom, most hospitals have insufficient numbers of intensive care beds, while in the United States there is enormous concern about the bed/patient relationship and the repercussions that this lack causes.^23,24^

In Brazil, the Ministry of Health recommends that all tertiary hospitals with more than 100 beds should have intensive care units, and that these units should account for at least 6% of the total number of hospital beds.^25^ However, the Ministry's statistics show that, while there was a 14% increase in the number of intensive care beds in the public health system from 1995 to 2000, the growth in the expenditure on such services was 77.5% over the same period.^26^ Thus, Brazil's public health system presents a scenario in which the emergency services are not in a position to cope with the whole demand from patients and there are insufficient numbers of intensive care beds in hospitals.^27^ In spite of difficulties in establishing what the true demand is, it can be said that there is a major shortage of intensive care beds in Brazil. Over a three-year period, an intensive care unit in a large public hospital in Recife, State of Pernambuco, was only able to provide intensive care for 67.6% of the internal demand and 61.3% of the external demand, thereby denying intensive care to around 452 patients over that period.^22^ The study in Recife shows again that, in Brazilian hospitals, the percentage of intensive care beds in relation to the total number of hospital beds is much lower than in other countries, particularly in relation to the United States, where the APACHE II system was developed.^20^ At the time of the original study^2^ the percentage of intensive care beds in relation to the total number of beds in hospitals in the United States was 5.6% and this figure had increased to 10% by 1992. In Europe this percentage ranged from 2.6% to 3.8%, and it was 2% in Japan.^3,5^

At our hospital, it is 2.5%, thus demonstrating the limited availability of intensive care unit beds. The characteristics of such a tertiary hospital school, which is a referral center for multiple trauma patients and highly complex procedures, underline the need for a larger number of intensive care unit beds. Because of this situation, many patients, particularly those in the over-12 h group, received medical attention in the origin location, while waiting for admission to the intensive care unit. Since the study started after the patients' admission to the intensive care unit, there was no interference in the admission criteria for patients. All of them stayed in the surgical unit, in the anesthesia recovery unit, and they received all of the services necessary from the anesthesia and surgical teams, which were responsible for the patient. This unit had monitors, ventilators, infusion pumps, catheters and the human resources needed for such services. In the event that there was more than one patient with an indication for intensive care unit services, the choice of which patient would be transferred first was made by the doctor from the anesthesia recovery unit, according to the hospital's procedures.

In spite of such measures, the possibility of deterioration in patients' clinical condition existed for as long as the transfer was not made. This situation therefore contributed towards greater severity of clinical condition. In this respect, the present study differed from other studies, particularly those conducted in the United States, where patients start to receive care in intensive care units almost immediately, with a very fast transfer from the emergency services or surgical the- ater.^9^ This partly explains why in Brazil there are higher APACHE II scores and higher mortality among such patient populations. This goes against one of the initial premises for APACHE II development in the United States: a good prognostic index must, as much as possible, be independent of treatment.^2^ The difference between Brazilian and North-American populations may also explain the discrepancy between the predicted and observed mortality among the patients in the over-12 h group.

The lack of beds that is found in emerging countries as well as in industrialized countries leads to conflicts of a technical, social and ethical order.^28-31^ Knowledge and detailed study of prognostic indices, their application possibilities and their limitations, can aid in the arduous task of squaring the great needs with the scanty available resources.

APACHE II was chosen for the present study because its data are simple, well-defined and reproducible, and they are collected routinely during the course of intensive care. Besides being known around the world, it is one of the most used tests in Brazil, and is one of the parameters that the Ministry of Health considers in its classification of intensive care units.^25^ Thus, there is a continuing need to evaluate possible interference in forecasting capacity. Many factors that interfere in the forecasting capacity of this index have already been identified: limitations within the index itself, diversity in the patient population, differences in the use and readiness of beds, selection criteria, and therapeutic interventions before the intensive care unit.^11,16,19,20,32^ Although the importance of delays and selection bias has already been mentioned a lot, none of these studies had the objective of evaluating these factors and quantifying them in relation to their influence on the difference between predicted and observed mortality.^14,19,20,32,33^

The present study made a detailed evaluation of one of the variables that could modify the accuracy of the APACHE II index: delays in the transfer to the intensive care unit. We chose to study patients who had undergone emergency surgery for two main reasons. Firstly, because delays among this kind of patient are easily measured: the counting of how long the delay is starts from the end of the operation, i.e. when the patient is removed from the surgical theater, and continues until when he or she is admitted into the intensive care unit. This is different from the situation of clinical patients, for instance, for whom the time when intensive care is indicated is hard to establish with accuracy. Secondly, because emergency surgery patients have a greater chance of being subjected to operative procedure without a guaranteed place in the intensive care unit, because of the urgency of the situation. This differs from patients undergoing elective procedures, for whom the availability of a vacant bed in the intensive care unit can be scheduled, or is the determinant of whether or not the procedure is performed. The fact that the operation is performed, even without the guarantee of a vacant bed in the intensive care unit, shows the deficiency in the healthcare system, because, on the one hand, there is no possibility of a transfer to another, similar hospital (in general, other hospitals are also operating above their capacity) and, on the other hand, if the procedure were not performed, this would imply the inadmissible omission of the only therapeutic possibility for these patients.

In comparing our results with others previously published, we saw that the average age was similar to the ages of patients in previous studies in Brazil (which ranged from 50 to 55 years)^13,20^ and in American and European studies (which ranged from 55 to 62).^2,12^ The latter also had male prevalence, similar to what was found in Brazilian studies.^13,14,20^ The present study presented a higher frequency of trauma patients than in American and European studies, but similar to what was found in other Brazilian studies.^2,11,14,19,20^ The main differences between the patients in the present study and those in other studies^1,20^ that evaluated the applicability of APACHE II were that in the present study there was:

- Higher percentage of traumatized patients;
- Higher average APACHE II score;
- Lower frequency of patients with APACHE II score of less than 10.

The standardized mortality rate found was lower than was found in Brazilian intensive care units in a collaborative study and in studies from other countries.^16,19,20^

At the time of admission to the intensive care unit, the two groups of the present study were similar regarding age, gender, diagnosis and severity (APACHE II). One of the reasons for this result is certainly the delay in admitting patients into the intensive care unit after the operative procedure. To characterize the delay, it was necessary to divide the patients into two groups, by setting a cutoff time between when intensive care was indicated (end of the surgical procedure – at the time of leaving the surgical theater) and when the patient was admitted to the intensive care unit. Theoretically, such a difference of time should not exist. Our reality, however, is that this type of delay is very common, and that it may have a negative influence of patient evolution. In the present study, we measured this delay and quantified its repercussion on patient evolution. Initially, we tried to correlate the delay with the mortality rate, imagining that as the delay increased, so would the mortality rate. This relationship was found to only apply during the first few hours of the delay, i.e. after a certain amount of delay (12 hours), the mortality rate stayed high and was no longer related to the number of hours taken for the patient to arrive in the intensive care unit. For this reason, the time of 12 hours after the operation was chosen as the cutoff point to define the patient groups.

Maybe the study that is most similar to ours is an article published in the United States in 2003 that evaluated the effect of delays in transfers to the intensive care unit, on the morbidity and mortality rate and on the cost of hospital stay, in a hospital with an intensive care bed/hospital bed ratio of 3%. Ninety-one patients were studied over a period of 16 months, and delayed admission to the intensive care unit was associated with increased morbidity and mortality, and with increased cost of the hospital stay. As in the present study, most of the patients were in the group for which there was a delay in admission to the intensive care unit (56 patients). However, differing from our study, these authors evaluated only clinical patients, for whom specific physiological criteria were adopted to establish the time when the intensive care unit was indicated, and the cutoff time used to define delay was 4 hours.^34^

Another important result from the present study was the difference in mortality rate between the two study groups. It is known that delay in providing specialized services may cause significant differences in patient evolution, particularly in acute situations. This is especially so when the best resources and treatments available are not applied at the right moment.^35-37^

The variables identified as mortality risk factors were: age, APACHE II, length of delay in transfer to intensive care unit, and length of hospital stay. Of all these factors, the only one that is easy to modify is the length of the delay in the transfer to the intensive care unit, and this factor is the most important one for the evolution of these patients. The delay in the transfer to the intensive care unit had a large effect, such that the mortality in the over-12 h group was significantly greater than the mortality in the up-to-12 h group.

If the delay in the admission to the intensive care unit reduced the forecasting capacity of the APACHE II system and increased the mortality rate, the question that arises is whether it was the prognostic index or the treatment that failed. This is a seemingly simple question that, to be appropriately answered, would demand more considerations and analyses than the present study set out to investigate.

The present study utilized an APACHE II score obtained upon admission of the patients to the intensive care unit. This would have been influenced by all services performed prior to the intensive care unit, and might have been very different from an APACHE II score for these same patients, obtained at the time when intensive care was indicated, i.e. immediately following the operation (a time that was not considered in the present study). Thus, a "polluted" APACHE II was evaluated, in accordance with the therapeutic measures adopted and the patients' own evolution following the operative procedure. We could not attribute the forecasting difficulty to a "flaw" in the index. On the other hand, the monitoring conditions and treatment offered as alternatives during the period before the patient was transferred to the intensive care unit (which were probably optimized to supply the patient's needs) was not analyzed in the present study, thus rendering discrepant any judgment of the treatment given before admission to the intensive care unit.

When there is a shortage of beds, the average degree of severity of patients' conditions increases.^30^ This study shows the situation of shortage of intensive care beds in a clear and objective way, at one of the largest teaching hospitals in the country, and also the degree to which the patients it cares for are in a condition of greater severity. This situation is far from being exceptional. In spite of data collection and publication difficulties in Brazil, it is not difficult to imagine, if only through professional experience and daily practice, that this situation is, unhappily, commonplace among major Brazilian public hospitals. Every day, the emergency services of these public hospitals experience the drama of the impossibility of transferring patients, because of the lack of vacant beds recorded by the duty administrators, and also the overloading of the referral hospitals. If, on the one hand, many ethical and social conflicts may appear, the reality of this situation forces us to create policies and differentiated rules to solve them, by using the available resources in an ethical, efficient and egalitarian way.

This situation is not ideal, although it is necessary in order to care for a larger number of patients that otherwise would not have any service. In this respect, the present study has been useful for quantifying the distortions, thereby enabling actions and measures for resolving or reducing them in a balanced way. We cannot take the radical approach of simply closing the doors of the service to many patients. Rather, we have to transform the available resources into a better condition, so as to improve the service standards.

Many measures can be taken, in seeking to resolve or reduce the problems identified and quantified in the present study:

1. Increase the readiness of beds in the intensive care units. Without a doubt, this is the most needed and effective measure. However, it usually runs up against the need for significant amounts of financial resources. Moreover, in some hospitals there are no appropriate places for intensive care units to expand into. Another limitation of this solution relates to the human resources needed. As a high-complexity service, there are not always enough professionals with sufficient specialization.
2. Decrease the number of services, with the aim of adapting the service level to the hospital's ideal operational capacity. Although this may be a theoretical alternative, it is unviable in practice, because there are no other hospitals to which patients can be referred. In addition, the reference hospitals are the ones that are in the best position to offer such services.
3. Planning and adaptation of the human resources and materials needed for optimizing services in the places where they are temporarily provided, i.e. alternative care facilities such as the serious patient units of emergency rooms, semi-intensive units in various departments and, in the specific case of the present study, the setting up of a service structure in the semi-intensive unit of the surgical center. One temporary measure of interest, when the financial resources or physical space for increasing the number of intensive care beds is unavailable, is the optimizing of human and technological resources in intermediate (semi-intensive) units, thereby qualifying them for service provision at a level similar to the level of intensive care units. The application of this measure in our hospital practically doubled the number of available beds for providing a service for this kind of patient.
4. Creation of norms and selection protocols for admissions to intensive care units and intermediate (semi-intensive) care units. Knowledge of the difference in mortality rate between the groups in the present study may alter the selection criteria for patient admission to intensive care units. The fact that this difference mainly occurs at higher scores of APACHE II (more than 21) may determine more specific criteria for attaining the objective of decreasing the mortality among patients whose admission into the intensive care unit takes longer (over-12 h group).

One of the limitations of the present study was the small number of patients studied. This limitation increased when we divided the patients in the two groups. This relatively small number of patients may, in theory, have had an influence on the results. The decision not to extend the study to cover a longer period, so as to increase the number of patients, took into account the significant and relevant results found and the possibility of implementing measures to improve the service conditions that, a priori, would benefit a larger number of patients. On the other hand, we cannot be sure that, if we had had a larger numbers of patients, and similar numbers in the groups, we would have had results any different from those that we obtained.

Continuation of the present study becomes important, in order to reevaluate the results, taking into account the changes made. The study should also be extended, so as to include the evaluation of other situations that have not yet been studied, such as:

- evaluation of patients for whom intensive care is indicated, but who are not admitted to the intensive care unit following the surgical procedure;
- evaluation of the selection criteria used;
- evaluation of admission delays among other groups of patients (clinical patients, or those undergoing elective surgery);
- evaluation of the therapeutic interventions used before admission to the intensive care unit.

The present study also shows that we need to create our own indices, or to modify the existing ones, with the aim of improving the forecasting capacity in relation to our patients, who have significant differences in relation to the patient populations on which the prognosis indices were validated.^32^ Thus, new studies that investigate the individual prognoses for patients in intensive care unit are necessary. These may supply important information on present-day intensive care. Investigation of prognoses within intensive care units may show up differences in the perception of the care given in these units, the patient selection, the real influence of technical resources on treatment results, and the application of successful experiences within similar patient populations. Only research and continuous experience of the use of these prognostic methods will enable improvement of our estimates. Equally important are the development and improvement of indices to evaluate not only mortality, but also the quality of life among these patients following their stay in the intensive care unit.

## CONCLUSIONS

The elapsed time between the end of the surgery and admission to the intensive care unit, for patients who had undergone emergency surgery, did not interfere in the length of stay in the intensive care unit and hospital. However, it significantly interfered in the prediction capacity of APACHE II, and in hospital mortality. The APACHE II system gave better mortality prediction for patients who arrived in the intensive care unit faster. The hospital mortality rate was higher among the patients whose admission to the intensive care unit after the surgical procedure took longer.
